# Renewed vision on pulmonary rehabilitation service delivery for chronic obstructive pulmonary disease management beyond COVID-19

**DOI:** 10.1016/j.cdtm.2021.01.003

**Published:** 2021-02-06

**Authors:** Shakila Devi Perumal

**Affiliations:** Cardiff University, Cardiff, Wales, United Kingdom

**Keywords:** COVID-19, Pulmonary rehabilitation, Health service design, Chronic respiratory disease, Integrated care

## Abstract

Pulmonary rehabilitation (PR) is a cornerstone management for chronic obstructive pulmonary disease (COPD). International respiratory societies defined PR is more than “just an exercise program”; it is a comprehensive care delivered by a team of dedicated healthcare professionals with a strong emphasis on long-term health-enhancing Behaviors. However, “Uncertainty” exists with varied reasons for the political and geographical barriers of referral, uptake, attendance, and completion of PR in both primary and secondary care. Besides, COVID-19 pandemic has sparked many global controversies and challenges on pulmonary rehabilitation service delivery. Post-COVID-19 guidelines emphasize on integrated care rehabilitation for patients with COPD. Thus, this concise review intends to understand the gaps in United Kingdom healthcare policies, practices, and PR services resources. To date, there is no clear consensus on PR integrated care model pathway to address the unmet needs, measure the health and social care disparities; adds to the disease burden of COPD. Based on the culmination of evidence, this perspective offers a theoretical framework of PR integrated service model, a pathway to deliver high-value personalized care to patients with COPD.

## Introduction

Chronic obstructive pulmonary disease (COPD) is a global health problem and predicted to be the fourth leading cause of death worldwide by 2030.^1^ COPD is the second most common cause of emergency admissions and fifth most common cause of mortality in the United Kingdom.^2^ COPD is an irreversible, progressive non-communicable chronic lung disease often linked with tobacco smoking or airborne exposure or inherited α-antitrypsin deficiency.^3^ Patients with COPD often present breathlessness, cough, physical inactivity, fatigue and frequent exacerbations.^4^ Besides, systemic co-morbidities, disease-specific exacerbations, poor self-management have adverse implications on patient and healthcare systems.^5^, ^6^, ^7^ The disease's unique complexity calls for integrating multidisciplinary professionals across all healthcare system levels to provide holistic and less fragmented care for patients with COPD.^8^

Pulmonary Rehabilitation (PR) endorsed as a “Gold Standard” non-pharmacological component of integrative respiratory medicine in the management of COPD.^9^^,^^10^ PR is defined as a comprehensive multi-component program that includes patient assessment, patient-tailored therapies of exercise, education, and psychosocial intervention designed for patients with chronic respiratory disease to manage their condition beyond the duration the program.^11^^,^^12^

A typical comprehensive PR components of care includes:

1. Inclusive assessment by an interdisciplinary team of specialists both in secondary and primary care providers.
2. Optimization of medical management, including supplemental oxygen.
3. Optimization of non-pharmacological management, including exercise training, physical activity, nutrition, psychosocial support.
4. Promote sustained self-management education, including smoking cessation intervention, vaccination, physical activity, energy conservation, nutrition, end of life etc.
5. Timely psychosocial assessment and support for cognitive impairment and behavioral change interventions.
6. Maintenance programmes and community support with relevant professional support.
7. Key performance measure of dyspnea, health status and exercise assessment and feedback by secondary and primary care providers.^11^,^12^

Despite confirmed benefits of PR, it is widely underutilized.^13^ Global variations exist between ideal and provided PR care, with regional heterogenicity in local healthcare systems.^14^ This review aimed to provide a concise overview of current gaps in United Kingdom PR healthcare policies, practices and resources with a resolution to design a theoretical framework COPD-PR service pathway.

## PR service gaps pre COVID-19

As per definitions, PR is more than “just an exercise program” or “chest physiotherapy” and warrants inclusion of multidisciplinary interventions to improve skills for “effective long term behavior change in patients with COPD”.^11^^,^^14^^,^^15^ Ideally, the PR program should be “One Shop Stop” offering standard comprehensive patient-tailored intervention such as medical, physical, psychological therapies and social care support to witness sticky “lifelong” health-enhancing behavior change. International PR guidelines recommend prescription of at least 20 sessions to achieve physiological benefits or more extended programmes to sustain the positive effects of health-related quality of life and behavior change.^16^ Evidence confirms at least more than 16 weeks of training deemed appropriate to improve aerobic fitness in healthy aged population.^17^ To date, there is no consensus on the optimal duration of PR, local resources and behavioral patterns for patients with COPD.^18^ In practice, most PR programmes in UK, have adopted a reductionist approach with shorter programmes of 6 weeks, 2 to 3 times per week delivered in various settings – primary, secondary, or community care positioned only on exercise training and education.^19^ Additionally, PR performance indicators are focused on functional walk test with least attention to daily living activities, or mental health, woefully missing the holism of multidisciplinary engagement.^14^^,^^19^

In recent years, increasing physical activity (PA) perceived as one of the crucial PR outcomes due to awareness of the detrimental effects of physical inactivity in patients with COPD.^20^ Higher daily physical activity levels are associated with potential health benefits and require early behavior change intervention partnership with multidisciplinary stakeholders for COPD patients groups B–D.^21^^,^^22^ Exercise vital signs (EVS) is a validated tool for screening and monitoring population-level PA.^23^ EVS could also provide data on social and behavioral domains on an individual level to tailor lifestyle behavioral interventions for patients with chronic lung disease. However, translation of evidence to routine remains challenging due to knowledge gaps in science and its physical activity benefits among patients and healthcare professionals.

While PR's immediate short-term benefits are undisputed, given the nature of severity of COPD, benefits including exercise capacity, symptoms, and quality of life wean as early as 3–4 months without any maintenance strategy.^24^ Long-term maintenance programme focused on self-management skills training is pivotal for patient engagement to trigger and sustain long term behavior change. Many centres deliver self-management only focused on virtual educational components and written action plans to patients with COPD. However, education alone is insufficient to achieve the goal of behavioral change.^25^ International expert group^25^ defines a self-management intervention for COPD as the structured programme with personalized multi-component goals to address patient motivation, engagement and skills development training for healthy behavior change. Self-management intervention's objectives require focus on personalized plans on physical, psychological and social well-being in COPD patients. Yet, to date, the transformation of self-management constructs to practice in PR programme is naïve.

In the United Kingdom, the National Health Service (NHS) and British Thoracic Society (BTS) quality improvement programs emphasize patient-centered self-management as the central tenet of PR programme initiatives.^26^ Few initiatives such as Expert Patient Programme (led by patients) and British Lung Foundation (led by exercise specialist) focused on delivering community support for exercise and dyspnea management in COPD patients. Taking a look at those initiatives, generally self-management programmes are focused on breathing retraining to combat exacerbations. In reality, beyond breathing issues, patients with COPD face a tremendous ambivalence towards adopting long-term healthy behaviors including compliance with pulmonary rehab sessions, smoking cessation, medications, oxygen therapy, physical activity, social networking and self-management techniques. These issues could be attributed to the misconception of PR's value, thus posing an economic challenge to NHS. Hence, PR programmes objectives should extend beyond symptom recognition for exacerbations. As COPD disease is associated with various psychosocial pathology, it is crucial to include ongoing professional support for health and behavioral interventions in PR programmes.

Behavior change plays a vital role in health promotion and is a significant challenge for current health promotion policy and practice.^27^ Behavioral interventions need to be tailored to patients and require continued, incremental, and focused sustainability efforts. Intervention strategies should draw attention to Albert Bandura theories – Social Cognitive Theory (SCT) and Transtheoretical Model (TTM) to address challenges of self-efficacy and readiness to compile with self-management recommendations prescribed in PR.^28^ Transformation of those theories to practice require skilled professionals to facilitate literacy sensitive, patient-centred interactions to identify barriers and facilitators for motivation, confidence and competence in patients with COPD. Traditionally, psychologist or psychotherapist is deemed mental health professionals to prescribe behavioral therapies. Precipitately, the role of mental health professionals is under-resourced in pulmonary rehabilitation programmes. For reasons little understood, the choice of mental health is at the patient's discretion in COPD care management. Universally, there is a lack of a consensus on what components constitute psychological and social constructs for COPD patients.

Past few years, the theme of public engagement was recognized as a crucial element for behavior change models underpinned by social practice theories, social networks and interactionism to reduce health care costs.^29^ New healthcare models in the United States, England and South Africa have promoted financial incentives to encourage healthy behaviors.^30^ Further evidence exists that contingency management (CM) programs that are incentive-based (vouchers or prizes) promotes effective health behavior change.^31^^,^^32^ Contrasting evidence confirms financial incentives for problematic behaviors is insufficient to elicit sustainable behavioral change.^33^ COPD net study^34^ adopted number of behavioral performance measures and shared decision making to design a personalized self-management care plan for COPD patients. COPD net study summons reform of local health policies and reconfiguration of PR service model in the UK for a culture of sustainable health behavior change.

To date, several challenges persist for successful implementation of a comprehensive PR.^35^ Besides, variation and lack of understanding of COPD phenotypes, extrapulmonary traits, disease severity, patient personal choices of healthcare complicate and challenges PR long term management in primary care.^36^^,^^37^ PR audit exposes only 13% of eligible COPD patients offered PR, and only 60% of patients were enrolled into PR within 90 days of referral.^38^ UK healthcare policies and system operates as a one-way traffic system where respiratory care specialist in secondary care initiates the majority of COPD referrals. Patient autonomy and accountability for their disease management has no structured constructs in health policy. Current PR care model for patients with COPD are not logical and brings along risks, waiting times and delays for the patient and duplication of tests causing additional costs to the health care system. Lack of structured inclusion of PR in primary care networks portrays a fragmented operational strategy that is a deterrent for implementing holistic person-centred or community-based approaches.

## Missing puzzles in PR care delivery

Presently in UK, PR care model for COPD focuses on “What is the matter” and the vision should be reverted to “What matters to the patients” to coproduce a simple, cost-effective and sustainable patient-centred care.^39^ Prominently, knowledge gap, barriers of local health policies, lack of resources for health care professionals training demands creative solutions for optimal PR care management. Some of the gaps are reliant at local-level health policies with heterogenous challenges, such as translation of clinical guidelines at the local level affected by local advocates and available resources, selection and implementation of health-related behavioral policies influenced by individuals health belief model.

In short, there is lack of consensus on what constitutes ‘success’ or ‘effectiveness’ for a standard model of PR integrated care to evade disparity in health care services for COPD population.^40^ Based on review, the author perceives significant barriers in implementing PR integrated care model suffers contingency plan on six process that includes: 1. Patient leadership in developing and implementing healthcare care policies for timely access to diverse care needs for early diagnosis (Accessibility). 2. Education of referral sources (medical and allied health professionals) with effective marketing strategies (Enrollment). 3. Tangible rewards for both patient and healthcare professional for personal and professional engagement (Governance). 4. Quality management system for patient and healthcare professionals' accountability at various levels of health care delivery (Adherence). 5. Robust performance management with multiple stakeholders (Quality Assurance). 6. Inclusion of patient perspectives for ongoing care provision (Sustainability). It compels us to rethink and reform our current PR operational systems to generate prepared clinicians, knowledgeable and confident patients.

Seeking to address and resolve health disparities, recent policy statement from international respiratory societies has several recommendations to close the theory-practice gap in PR.^41^ The key recommendations include: 1. Inclusion of formal training and ongoing professional development in the national curriculum for physicians and allied health professionals involved in respiratory care. 2. Increase public awareness by developing patient education materials on PR's process and benefits in multiple formats aligned to the region's linguistic and cultural needs. 3. Increase the PR capacity by commissioning new model of evidence-based, sustainable integrated PR programmes in primary and community care. 4. Ongoing monitoring on the key performance measures to ensure quality assurance.

## UK health care policies and systems response

In 2010, COPD consultation group drafted a summary on commitment to expand and integrate PR interventions with cardiac rehabilitation programme. In 2019, NHS Long term plan^42^ recognized “chronic respiratory disease” as a national clinical priority, and healthcare systems need to innovate care model tailored to the evolving needs of diverse communities. Following, NHS improvement plan commissioned to establish Integrated Care Systems (ICS) across the UK to expand PR services putting ownership on primary care networks. The objective was primarily to: 1. increase the PR's referral rates from 13% to 60% by 2023; 2. Patients digital access to their medical records from 2020/21; and 3. Expansion of mental health services with integration of physical activity by 2023. Additionally, nature-based social prescribing recognized as a crucial care element of GP contract to improve the efficiency and effectiveness of primary care services for psychosocial well-being of local communities.

In alignment with NHS Long term plan, the BTS position statement on Integrated Care Model^43^ had identified two significant inconsistencies in PR service delivery for COPD patients: 1. Access and delivery of post-hospitalization PR; 2. Adherence to guidelines and quality standards. Subsequently, actionable steps were targeted on: 1. continuous digital education support for patients attending pulmonary rehabilitation; 2. Integrated digital decision support; 3. Incentivizing cross-organization working system design; and 4. Quality assurance of clinical data collection and audit process. To support the action steps, two key initiatives such as NHS long-Term Plan and National GP incentive scheme were commissioned to promote and build PR capacity. In 2018, pulmonary rehabilitation services accreditation schemes (PRSAS) was launched to monitor the PR quality assurance by the royal college of physicians.^44^ Yet this scheme is predominately recognized only in England and it is in nascent stage. Some perceived barriers for PRSAS were the funding, resources, training and integrated constructs for PR services.

Despite those actions and initiatives, recent PR clinical audit interim report^45^ revealed only two hundred and twenty-three services participated in England (*n* = 194), Scotland (*n* = 18) and (Wales *n* = 11) for a UK population of 66,796,807 (mid-2019), shedding light on the uneven distribution of PR programme nationwide. Another report confirmed 14 different ICS model across England are not meeting the expected reductions in hospital admissions^46^ due to varied reasons: 1. Lack of patient perspectives; 2. Low multidisciplinary working partnership at primary care; 3. Lack of SMART outcomes measures. Moving forwards, ICS model should involve primary care networks and have a shared vision to tackle personal, behavioral and social care for patients with COPD.

## COVID-19 and pulmonary rehabilitation

COVID-19 has brought unprecedented challenges and unparallel opportunities for innovations in global health care delivery models, including pragmatic pulmonary rehabilitation services.^47^ The patient-centred care and experience, a central precept of the NHS long-term plan, is now made mandatory to build a resilient care system that prioritizes value, accessibility, satisfaction, and, most importantly, outcomes. These health imperatives have placed an undeniable demand on telehealth and virtual solutions that can no longer be set aside. Usage of telehealth and virtual care has already risen steeply in the last eight months. Studies confirm remotely supported PR with telecare is non-inferior to traditional centre-based PR.^48^^,^^49^ The swift shift toward digital delivery provides a clean slate for clinical leaders to understand their patient population's current clinical condition, experience and staff needs for a sustainable health system.

Presently, with ambiguous promises on new vaccines, we are entering into new challenges of Post-COVID-19 rehabilitation. Recent BTS update on “business as usual” has documented guidance on revamping PR with alternative business solutions (digital) to ensure the best quality of post COVID-19 rehabilitation. Guidelines for post-COVID-19 rehabilitation emphasize an integrated multidisciplinary approach involving medical doctors, physiotherapists, and psychotherapists.^43^^,^^50^ Many UK centers yet to establish these services and optimal care management remains unclear. Though there was a swift cultural change of virtual connections of video calling for patient consultation, still there were some unknown barriers for a consistent, integrated care model to deliver consistent and agile PR services. Arguably, it is unknown if COVID-19 is a percussor for a cultural change of technology-based solutions for sustainable care amidst the ambiguity of cybersecurity and integrity of health care data.

## Pragmatic solution

In the 20th century, patient-centred care (PCC) concepts had permeated the healthcare systems with a cultural shift to care delivery focused on patient satisfaction and outcome.^51^^,^^52^ Evidence on patient-centered care confirms decreased healthcare utilization, improved patient compliance and quality of care.^53^ In this modern era, the health care system is subjected to continuous transformational shifts from volume- to value-based care. Seeking to overcome the volume issues, integrated care, and patient care coordination would be pragmatic solutions to deliver equitable PR services to diverse communities.^54^ Currently, there is no consensus on sustainable performance indicators for integrated care systems. Moving forward, reconfiguration of pulmonary rehabilitation services in primary care should include elements of live, personalized care programme, agile staffing models, continual outcome assessment and patient engagement with parity to diverse communities.^55^ Integration of artificial intelligence (AI) and augmented reality (AR) would be an intuitive way of reconfiguring pulmonary rehabilitation care delivery to target personalized care and patient engagement.^56^

Integrated telerehabilitation would be cost-effective on workforce demands for diagnostics and therapeutic interventions to target the right care, at the right time, for the right people. Based on pragmatic evidence, personalized COPD management needs to be tailored on pulmonary, extrapulmonary and behavioral treatable traits.^57^ PR is an “integrative model approach” to attain quality, accessibility, cost-effectiveness and patient participation.^58^^,^^59^ Studies confirm remotely supported PR with telecare is non-inferior to traditional center-based PR.^48^^,^^49^ Most PR interventions could be provided in primary care adopting telemedicine solutions (AR & AI) and chronic care model to improve timely access, enrollment, and engagement for COPD patients.^60^, ^61^, ^62^ A distinction between emotional and functional dimensions of quality of life may improve the design and evaluation of integrated health care programmes in patients with COPD.^63^ A sustainable, high-quality, evidence-based PR care at a sensible cost, demands technology-enabled collaborative partnership between public, private and voluntary charity organizations to deliver innovative models of multi-component care services for patients with COPD.^64^^,^^65^

## Proposed theoretical framework – RAPRIS model

**Fig. 1:**
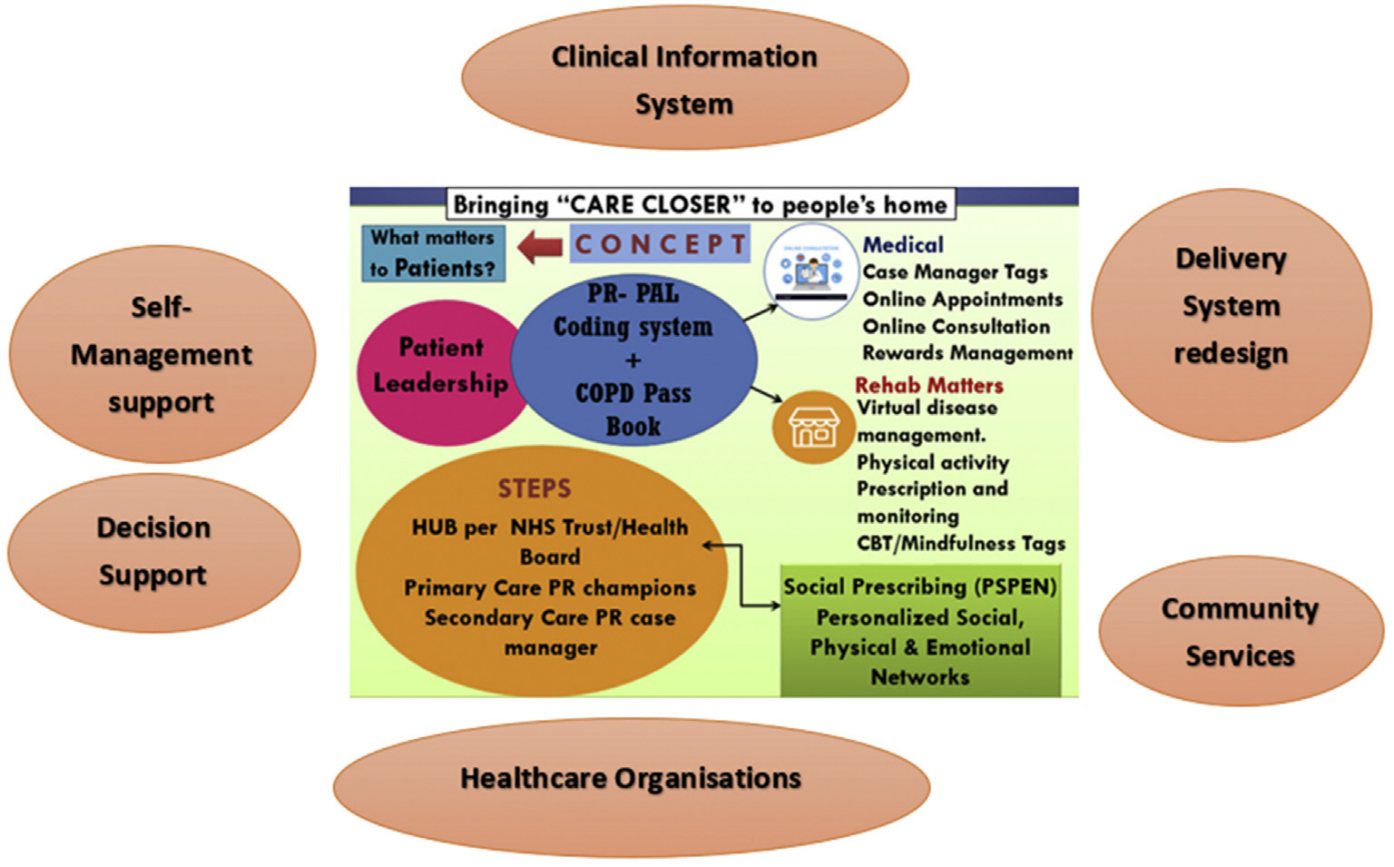
Chronic care model and pulmonary rehabilitation service design (from Shakila Devi Perumal with author's permission).

From the analysis of evidence, healthcare policies and perspectives, it is crucial to be open for a disruptive innovation of an autonomous, opportunity-driven service model to reform the healthcare system. In recognition of knowledge and geographical gaps, it would be intuitive to harness both AI and AR in a primary care setting to create a community of COPD population practices. Thus, the author proposes a theoretical Framework – “Rapid Access Pulmonary Rehabilitation Integrated Services” (RAPRIS) model at every primary care centre in UK bringing care closer to people's home (Fig. 1). RAPRIS model is based on theoretical frameworks of learning^66^ and serves as a platform for patient-centred outcomes service and knowledge hub for healthcare practitioners involved in care delivery for COPD patients.

The global objective of RAPRIS model is to alleviate health and social care disparities with integrated technology-enabled solutions to local communities for sustainable patient empowerment and quality care. In RAPRIS model, each primary care hub will be governed by Primary Care Champions (GP/Nurse/Allied health professionals) for patients with COPD. Mental health professionals (psychologist or psychotherapist) and social prescribing link workers will be commissioned on a ratio 1:1000 population at every primary care hub to address social and mental health needs of COPD patients. A Patient Care Coordinator (PCC) will be commissioned at every primary care hub and acts as a case manager with support of “PR Pal” (Virtual). Besides, a secondary care respiratory case manager will be commissioned at every hospital to prevent duplication or fragmentation of referrals and enhance health care delivery's integrity at the right time to the right people. Block funding model will be adopted to commission private RAPRIS clusters per clinical commissioning group (CCG) to increase PR capacity in primary and community care settings.

The novel features of this RAPRIS model includes virtual platforms of “Team-based learning (TBL) knowledge hub”, “PR Pal”, “Behavioral Deposit Scheme” and digital telehealth using AI and AR. Virtual team-based learning (TBL) knowledge hub aims to create a community of practices in COPD care delivery for both patients and healthcare professionals. Self-referral is promoted for patient leadership on their disease and “Behavioral Deposit Scheme” would be incorporated to sustain patient and family engagement in COPD care plan. Digital telehealth will support diagnostic – disease profile, care coordination, decision support, patient education and professional training updates. Patients will also be provided with open access to E-health record and Digital COPD Pass Book, color-coded based on disease-specific and behavioral factors.

### Operations of RAPRIS model

**Fig. 2:**
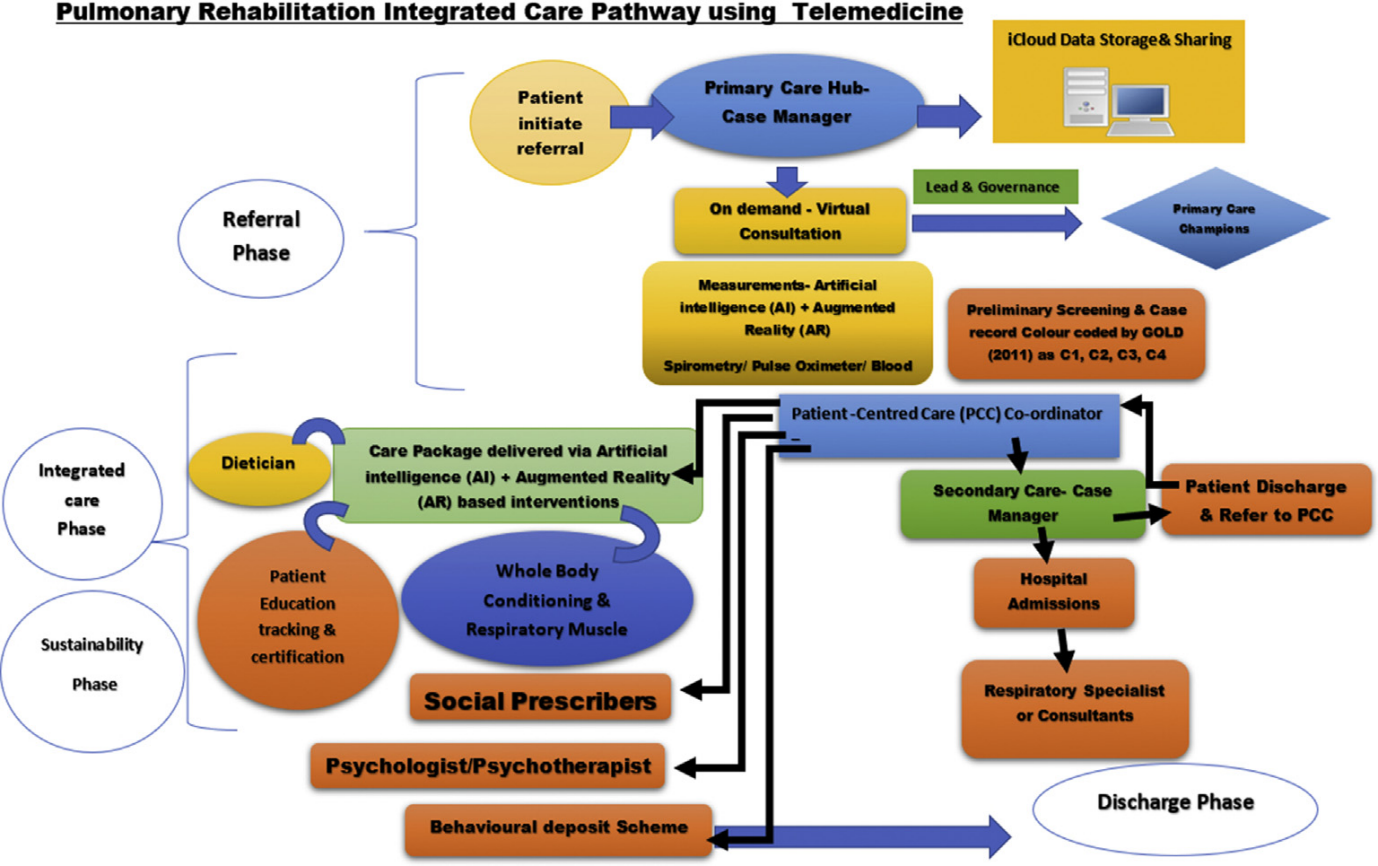
Pulmonary rehabilitation integrated care pathway using telemedicine (from Shakila Devi Perumal with author's permission).

The theoretical framework of RAPRIS service design (Fig. 2) includes four phases: 1. Referral Phase; 2. Integrated care phase; 3. Sustainability Phase; 4. Discharge Phase.

#### Referral Phase (0–Week 2)

This phase is driven by patient leadership via E-health Hub platform with enhanced features of AI and AR. Patient with symptoms of COPD initiates self-referral for virtual on-demand consultation to PCC. Following, PCC will process the referral within 48 hours to primary care champions for initial virtual consultation to determine the symptom, motivational barriers and diagnostic profile of the patient. Primary care champions will conduct initial virtual consultation to formulate patient medical record (PMR). Major parameters include diagnostic measurements, assessment of patient disease profile, prescription and goals of the COPD care plan, social care and motivational factors. PMR will be processed through electronic patient data management and triage system (EPMTS). All patient information will be password protected and stored in iCloud with a consented access to relevant healthcare professionals.

Patient data will be used to color-code digital COPD passbook to personalize patient care needs. Color coding would enable healthcare providers to access more accurate and detailed clinical information securely to inform clinical decision making. E-health platform also provides customized information to patients on lab results, clinical appointment reminders, self-scheduling, secure e-mail with providers, prescription refills, and educational content. Patient access to their health care diagnostic information will stimulate accountability and lifelong engagement in their health management decisions.

#### Integrated care phase (Week 3 to Week 30)

During this phase, PCC will review medical and rehabilitation needs via virtual behavioral and motivational screening surveys to identify patient self-selected health enablers for treatment, education and self-management to tailor care package. Patients would receive a care package notification within 48 hours following their initial virtual consultation. PCC will ensure ongoing care coordination including liaison of medical and social support, virtual follow-up consultation and case review every three months, and continuous virtual “team-based education” for patients. Virtual consultations and interventions promote health belief model bringing rehabilitation care in patients home saving time, money, unnecessary travel and discomfort.

#### Sustainability Phase (Week 3–Week 52)

In this phase, behavioral interventions based on Social Cognitive Theory (SCT) or Transtheoretical Model (TTM) or Cognitive Behavioral Therapy (CBT) will be designed and delivered over the E-health platforms for various behaviors such as physical activity, alcohol, smoking, anxiety and depression. To improve patients' self-efficacy in COPD management, “Behavioral Deposit Scheme” will be introduced to comply with appointments and self-management programmes.

#### Discharge Phase (Week 52)

In this phase, PCC would assess significant performance outcomes in three domains: physical, psychological and social to initiate a discharge or transfer to secondary or tertiary care settings. On the decision of discharge, a patient would be given lifetime access to virtual PR self-management programmes. Discharged patient data would be retrieved by local primary care hub for ten years from the day of initial patient contact.

## Conclusion

People with COPD has multifaceted barriers to access timely pathway of high-value care from diagnosis to pulmonary rehabilitation. NHS healthcare systems are often pressurized by demand exceeding the system's capacity. The hypothetic framework of RAPRIS model and recommendations are the culmination of ideas gathered from the literature evidence. Engagement of health technology solutions (AI & AR), together with the application of decentralized governance (PCC) in primary care settings and risk stratification measures would be an intuitive strategy for effective holistic COPD management beyond COVID-19 pandemic. Success of proposed theoretical framework on RAPRIS model demands a project team with drive, autonomy, expertise to attract investment from executive healthcare decision-makers to pilot and embed this model in current healthcare systems.

## Conflicts of interest

None.
